# Genetics and voice production in childhood and adolescence – a review

**DOI:** 10.1016/j.ijpam.2021.02.005

**Published:** 2021-02-19

**Authors:** Mette Pedersen, Anders Overgård Jønsson, Christian F. Larsen

**Affiliations:** a Medical Centre, Østergade 18, 1, 1100, Copenhagen, Denmark; b University of Copenhagen, Faculty of Health and Medical Sciences, Blegdamsvej 3B, 2200, Copenhagen, Denmark; c Copenhagen Business School, Solbjerg Plads 3, 2000, Frederiksberg, Denmark

**Keywords:** Voice production, Development, Adolescence, Genetics, Childhood, Puberty

## Abstract

Adolescence is a challenging time of change in voicing, normally and in pathology. An increased focus on voice production in relation to genetics can expand our knowledge of the onset of puberty and voice change. Our aim with this review was to connect research of genetics to voice production in adolescence. We need further understanding of the developmental background of voice in childhood and adolescence, because many genetic multi handicaps include voice production. Genetic development related to voice production was the focus in a search made by the Royal English Society of Medicine, with only a few results. We supplemented with references to genetic studies of adults and animals as well as adjacent areas of voice production. The genetic development of voice production is steered from the hypothalamus probably related to growth hormone. The genetic voice production in adults form the basis for understanding development. Some research results were found related to the pubertal steps. The findings are important in the future, using advanced voice analysis and artificial intelligence methods in patients with Multi handicaps.

## 1 Introduction

Adolescence is a challenging time when voices change normally and pathologically . Knowledge of hormonal changes based on genetic stimulation should be updated. Therefore, we present the results in an overview of the voice production in relation to genetics.

Also, measurements of acoustics of voice production is getting broader for bigger amounts of data, e.g. the technical methods for measuring voice production include highspeed films combined with analysis programs like Glottal Analysis Tools (GAT). Convolution networks analysis of highspeed films with 4000 pictures per second is used in the clinical setting as well as optical coherence tomography [[1], [2], [3], [4], [5]]. These methods with high amounts of input information should be related to better and more exact methods of genetic measurements in childhood and during puberty, to help many multi handicapped with a better communication possibility [6]. Many other aspects could be referred to.

The aim of the overview is to discuss the possibility for a relation between measurement of exact voice production and development at the level of genetics. Studies of voice production have earlier been made, defining prepubertal, pubertal, and post pubertal voices but without genetic aspects [7].

Voice measurements should be an integrated part in the research of developmental pathology e.g. genetic malformations including cochlear implants, from where much understanding can be gained [8]. It should supplement the very arbitrary definition of self-evaluated pubertal voice break used in many connections [9].

## 2 Methods

The search for voice production and genetics development made by the library of the English Royal Society of Medicine (RSM) at the end only included a few hand searched papers for the last 10 years; only a few of them were related to childhood and puberty. English language or foreign language article with an English abstract, human studies, and conference abstracts in Embase and Medline were searched. Since the amount of papers that included voice production studies, was rather small, we included our own intensive search of other papers, often based on reference lists, and in studies of other subjects - that could be relevant for understanding genetics in childhood and puberty also without voice measurements.

## 3 Results

### 3.1 Basic results of genetic studies

The brain development of voice production related areas in childhood and puberty is genetically structured. At the beginning the activation of the hypothalamic hypophysis gonad axis is a result of a complex network of genes, neurotransmitters, and neuronal interactions in the hypothalamus. It all begins from the nasal placode wherefrom GnRH (gonadotropin releasing hormone) neurons migrate to hypothalamus [10,11].

**Fig. 1:**
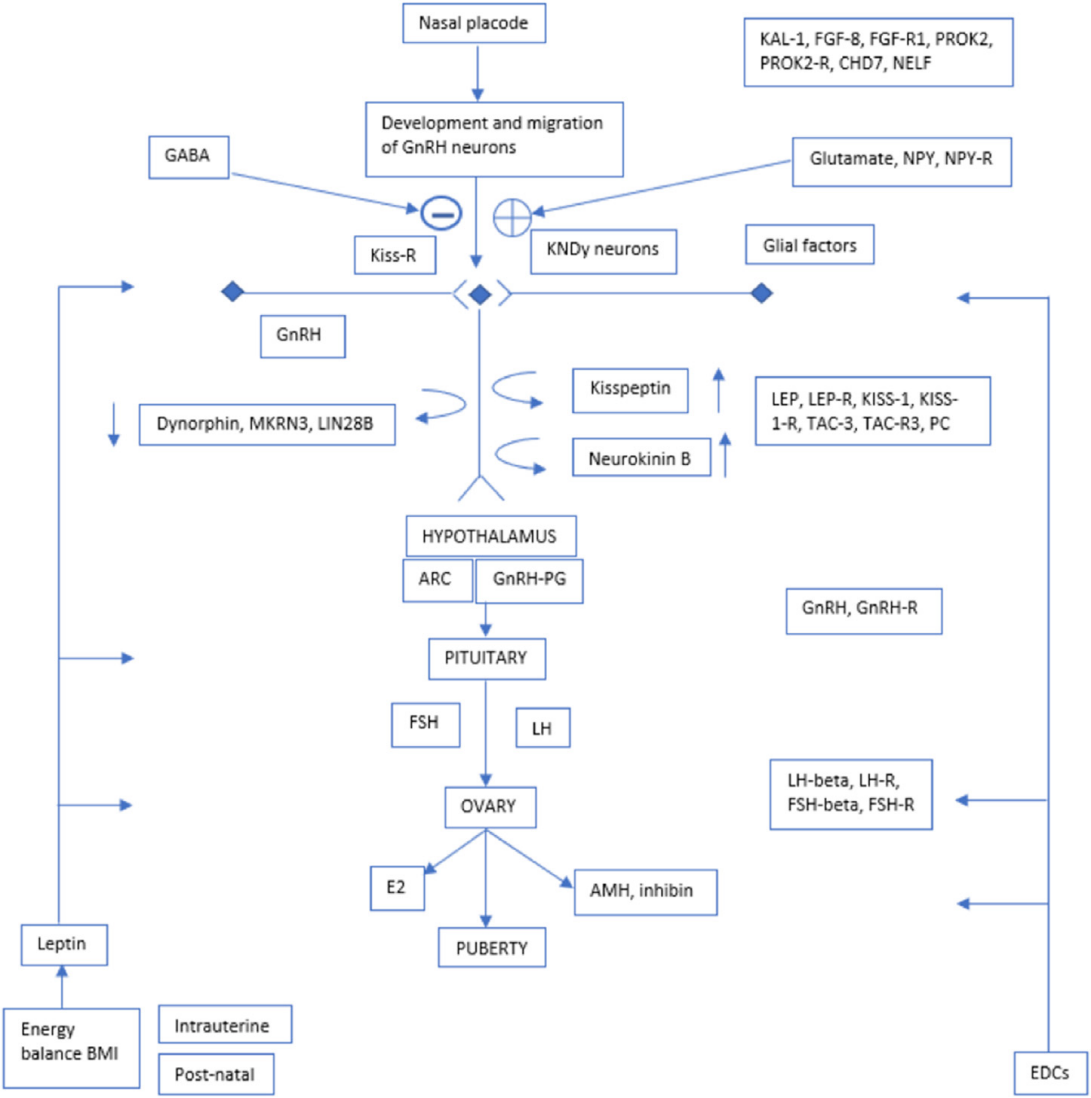
Presents an overview of the genes involved in puberty regulation with hypothalamus in the center. The development starts from the nasal placode in the fetus with development and integration of GnRH neurons (gonadotropin releasing hormone expressing neurons).

Fig. 1 shows the central activation of GnRH neurons by leptin, considered necessary for normal pubertal development. The nasal placode, and development of GnRH neurons have been considered a causal mechanism as stimulating factors on KNDy neurons (Kisspeptin, neurokinin B, dynorphins). The genes involved are included in the figure. The neuropeptide alfa-MSH plays a key role in energy homeostasis by mediating the action of leptin and may have a central role to the metabolic control of puberty. From the hypothalamus, at the medio basal area, the arcuate nucleus (ARC), the pituitary gland is stimulated, also by the GnRH-PG (Gonadotropin Releasing Hormone-Prostaglandin), the reaction depending on the GnRH-R – (Receptors). Follicle stimulating and luteinizing hormones stimulate the ovaries in girls, and in boys the production of Leydig-cells’ production of testosterone, stimulating puberty. In girls the Anti-Müller hormone (AMH) for egg reserve and inhibin level of hindering FSH production in the pituitary are regulated in balance with E2 (estradiol), stimulating puberty. Extrinsic factors (EDC) and body mass index (BMI) have an impact on the development [6].

An overview of genetic voice disorders was made in Phoniatrics 1 [6]. The main reason for this work was that it is now understood that genetic pathology of human development mostly involves voice production. The importance was underlined by Sataloff [12].

There is a genetic mix of research in the field, genetic factors influence vocal quality development but only narratively described in the literature [12]. Genetic correlations indicated shared etiologies in both sexes between puberty timing, body mass, and voice. Renes et al. [13] describe the dependency of growth hormone on normal functions of growth receptor hormones based on a gene on chromosome [5]. Gonadotropin releasing hormone (GnRH) is active in many connections as described by Forni et al. [14] The importance of understanding the relation between GnRH and among others fibroblast growth factor was described by Cho [11].

Lardone et al. [15] commented that voice break is a landmark of advanced male puberty in genome wide association studies and have revealed that pubertal timing is a highly polygenetic trait. They refer that although voice breaks are easily recorded in large cohorts, it holds quite low precision as a marker of puberty, 29 significant and independent single nucleotide polymorphisms were extracted associated with age at voice break. In contrast godanarche and pubarche are earlier and clinically well-defined measures of puberty onset.

The articulatory loop refers to the phonological loop with direct cortical control of the vocal fold musculature and the consolidation of an auditory-articulatory circuit, encompassing auditory areas in the temporoparietal junction and prefrontal and motor areas in the frontal lope, the connection between the anatomical and genetical understanding is still to be developed [16]. The definition of puberty stages 1–5 is referring to Marshall and Tanner [17,18]. In a large survey of puberty by Sultan et al., [10] voice production is not discussed at all. The author states that puberty cannot be perceived as a solitary event, they discuss the basic genetic changes. It is clear that genetic timing and e.g. age of menarche are relevant, Sultan et al. [10] underline that the onset of puberty is determined by genetic heritability and neuroendocrine factors (modulated by general health, nutritional adequacy, exercise and environmental chemicals). The understanding of voice production is also related to research on transgenders; Styne [19] has an overview of adolescence with the aspect of transgenders without commenting voice development. The forms of nuclei in arcuate- and antero-ventral- peri-ventricular nuclei account for the differential behavior of the hypothalamic-pituitary-gonadal axis.

### 3.2 Basic voice production measures

There was a change of understanding of the human voice with the vowel research made by Peterson and Barney [20]. Much has happened since. Fant [21], in his book of acoustic theory of voice production and Carlson and Fant [22,23], discussed the isolated vowels. Formants and resonance are discussed [24,25]. Henick and Sataloff [12] refer that the mutational voice is between 12 and 14 years of age and that the vocal folds in males at 16 years of age are 18–24 mm long with a fundamental (Fo) of 130 Hz. In girls, 16 years of age the vocal folds are 15–20 mm long and fundamental frequency (Fo) is 220–225. At 6–12 years the vocal folds have two layers, at 16 years the vocal folds have three layers, which is documented with optical coherence tomography [26].

Puberty stages 1–5 of average development [17,18] is not the same as the beginning of the various parts of puberty: adrenarche (of production DHEAS and androstenedione in the adrenals), thelarche (breast development), menarche (beginning of menstruation), pubarche (pubic hair development), godanarche (secondary sex characteristics). These to some extend genetically defined beginnings have till now not been related specifically to voice parameters.

Table 1 and Table 2 show the puberty related voice parameters, related to hormonal development divided in prepubertal – pubertal and post pubertal parts based on age [7], the pubertal stages can be extrapolated [17,18]. Fundamental frequency during reading of a standard text and the lowest tone as well as voice range during speaking are of interest in both genders. Androgens, testosterone, and serum hormone binding globulin (SHBG) are of special interest. Estrogens E1 and E2 should be noted. Change in DHEAS marks adrenarche.

**Table 1:** Hormonal, pubertal, and vocal parameters for **boys**.

Age	Years	8.7–12.9 y	13.0–15.9 y	16.0–19.5 y	Annual change (%)
No of boys		19	15	14	
Serum testosterone	n mol/l	0,54	10,5	18,9	68
Dihydrotestosterone	n mol/l	0,18	1,21	1,57	37
Free testosterone	n mol/l	0,007	0,14	0,33	77
Sexual hormone binding globulin	n mol/l	134	66	45	−16
Delta 4 androstenedione	n mol/l	0,54	1,17	2,5	24
Dehydro epi androsterone sulfate	n mol/l	1400	4100	5900	25
Testis volume	ml	2,3	13	20	36
Fundamental frequency	Hz	237	184	125	−11
Semitones in continuous speech	Semitones	3,7	4,8	5	3,9
Phonetogram area	cm^2^	19	28	34	9,2
Lowest biological tone	Hz	158	104	72	−12

Results are presented as geometrical averages. Groups: pre-pubertal (8.7–12.9 years), pubertal (13.0–15.9 years), post-pubertal (16.0–19.5). The annual change in %. Cm^2^ conversion factor: 1 cm^2^ = 32 semitones x dB(a). Semitone range is measured from the lowest frequency to the highest frequency in number of semitones.

**Table 2:** Hormonal, pubertal, and vocal parameters for **girls**.

Age	Years	8.7–12.9 y	13.0–15.9 y	16.0–19.8 y	Significance
Total number		18	12	11	
Oesterone (E1)	p mol/l	57	104	123	∗∗
Oestradiol (E2)	p mol/l	73	135	108	
Total testosterone	n mol/l	0,5	0,76	0,94	
Free testosterone	n mol/l	0,006	0037	0,009	
Oesterone sulfate (E1SO4)	p mol/l	732	1924	2342	∗∗
DHEAS	n mol/l	3210	3700	7200	∗∗
Androstendione	n mol/l	1,44	3,28	3,43	∗
Sex hormone binding globulin (SHBG)	n mol/l	153	130	123	
Menarche		+4	+9	+11	
Pubic hair stage		1–4	2–5	4–6	
Mamma development stage		1–4	2–5	5	
Fundamental frequency in continuous speech	Hz	256	248	241	
Semi tones in continuous speech	Semitones	3,7	4,2	5,2	∗∗
Semi tones in singing	Semitones	23	30	38	
Phonetogram area	cm^2^	17,3	21,8	28,3	∗∗
Phonetogram lowest tone	Hz	166	156	145	∗
Phonetogram middle tone	Hz	429	409	413	
Phonetogram highest tone	Hz	1136	1105	1263	

Results are presented as geometrical averages. Groups: pre-pubertal (8.7–12.9 years), pubertal (13.0–15.9 years), post-pubertal (16.0–19.8). The relative standard deviation range: 11%–140%. Significance of the differences between the groups: ∗∗ = p < 0.01; ∗ = p < 0.05. Cm^2^ conversion factor: 1 cm^2^ = 32 semitones x dB(a). Semitone range is measured from the lowest frequency to the highest frequency in number of semitones.

The sex hormone receptors in vocal folds have been focused upon by Nacci et al. [27] but were seldom found. The authors speculate that the changes of voice according to gender throughout life might be linked with a different expression of some genetic growth factor in the laryngeal tissue and that this expression might in turn be influenced by hormonal variation.

### 3.3 Some specific results in animals

There is a mix of genetic research in the field: animal studies, new-born studies, pubertal pathology studies, among others. Genetic factors influence vocal quality development but only narratively described in the literature although speech and language development is described [12,28]. The development of GnRH is important for the functional reproductive systems in vertebrates including PAX6, SOX2 and FOXG1. Kotler J and Haig [29] focus on anthropology in the difference between vertebrates. Based on studies in primates, Aboitiz [16] propose a continuous evolution for the auditory vocal apparatus. It is a problem that a lot of research on genetics and hormones is on primates/non-humans, which means that even if some results are common – when it comes to human voice, they can probably not be used.

### 3.4 Specific results in humans

Day F et al. [30] recognizes the biological genetic mechanisms and timing of puberty as important. In the recent large-scale genome wide female developmental study, 389 statistically independent signals were found distributed across all 23 chromosome pairs. According to Hollis et al. [31], in a male study, 76 independent genetic signals for male puberty was described. The authors found that genetically the voice break in boys was related to menarche in girls. Day et al. [32] also found 2 genes reportedly disrupted in rare disorders of puberty: LEPR and KAL1. A cluster of imprinted genes on human chromosomes 15 and 14, genetic variants in DLK1 are associated with menarche timing in girls and voice break in boys and pathology thereof.

The genetic and epigenetic approach to puberty is probably important for future aspects as examples in twins have shown for specific hormonal disorders e.g. SOX3 [33,34]. Schriberg et al. [35] have updated overview of percentages of neurodevelopmental disorders of speech/motor-speech.

Interesting is a study from Sato et al. [36] showing that the vocal fold mucosa, unphonetic, without voice, two cerebral palsy children (7 and 12 years old) did not have a vocal ligament, lamina propria appeared as a uniform structure, vocal fold stellate cells synthesized fewer extracellular matrix substances such as fibrous protein and glycosaminoglycan.

Aguiar-Oliveira et al. [37] present a study on humans with IGHG gene-type 18 owing to a mutation of GnRHr with severe reduction of growth hormone which resulted in voices that were high-pitched. Valenca [38] and their group also found in untreated isolated growth deficiencies that most voices had higher formant frequencies than normal, with a prepubertal acoustical structure. De Andrade [39] and their group showed that voice problems in patients with growth deficiency could be improved with the voice therapy of semi occluded vocal tract training.

## 4 Discussion

Some interesting results were found. In the pediatric literature, voice break is the parameter used for the change of voice production in puberty [9]. The pubertal genetic development itself seems to be rather well understood (Fig. 1) That might be the reason for the difficulties of comparing genetics to the pediatric development-since there are several voice production parameters to take into account: a part from a god measurement of fundamental frequency that includes registration in reading or counting, also measuring of the lowest tone and widening of the fundamental frequency range during reading – at best in semitones with geometric averaging of Hz (Table 1, Table 2). Other parameters include changes in formant structures [8,24,25].

From a genetic point of view – the aim should include the pathological genes involved in the specific Multi handicap syndromes first – and with GAT measures on highspeed films and AI of neural networks, to combine many measurements of each syndrome to find a pattern of genetic pathology related to voice production. These aspects seem to be necessary in society at the time where some genetic treatment aspects of multi handicap syndromes are under way.

## 5 Conclusion

An online search was made in Medline and Embase and a hand search of the Royal Medical Society, UK with modest results. Many indirect relevant studies of voice production development and genetics especially in adolescence were found in reference lists. It was shown how development of voice production is connected to genetic development. This is of main value for understanding pathology. New areas of voice production diagnostics are of importance. The genetic development of voice production is regulated from the hypothalamus probably related to growth hormone. Especially in pathology, genetic multi handicaps clients might get better help when combined genetic and voice production measures are used.

## Author contributions

Conceptualization: MP. Data curation: MP, AOJ. Formal analysis: MP, AOJ. Funding acquisition: MP. Methodology: MP, AOJ Visualization: MP, CL, AOJ. Writing – original draft: MP. Writing review & editing: MP, CL, AOJ.

## Funding

This research did not receive any specific grant from funding agencies in the public, commercial, or not-for-profit sectors.

## Ethical Statement

Hereby, I Mette Pedersen consciously assure that for the manuscript **Genetics and voice production in childhood and adolescence– a review** the following is fulfilled:

1) This material is the authors’ own original work, which has not been previously published elsewhere.
2) The paper is not currently being considered for publication elsewhere.
3) The paper reflects the authors’ own research and analysis in a truthful and complete manner.
4) The paper properly credits the meaningful contributions of co-authors and co-researchers.
5) The results are appropriately placed in the context of prior and existing research.
6) All sources used are properly disclosed (correct citation). Literally copying of text must be indicated as such by using quotation marks and giving proper reference.
7) All authors have been personally and actively involved in substantial work leading to the paper, and will take public responsibility for its content.

I agree with the above statements and declare that this submission follows the policies as outlined in the Guide for Authors and in the Ethical Statement.

## Declaration of competing interest

The material is not published previously and will not be submitted for publication elsewhere.

No conflict of interest relevant to this article were reported.
